# AcmD, a Homolog of the Major Autolysin AcmA of *Lactococcus lactis*, Binds to the Cell Wall and Contributes to Cell Separation and Autolysis

**DOI:** 10.1371/journal.pone.0072167

**Published:** 2013-08-08

**Authors:** Ganesh Ram R. Visweswaran, Anton Steen, Kees Leenhouts, Monika Szeliga, Beata Ruban, Anne Hesseling-Meinders, Bauke W. Dijkstra, Oscar P. Kuipers, Jan Kok, Girbe Buist

**Affiliations:** 1 Department of Molecular Genetics, Groningen Biomolecular Sciences and Biotechnology Institute, University of Groningen, Groningen, The Netherlands; 2 Department of Medical Microbiology, University of Groningen, University Medical Center Groningen, Groningen, The Netherlands; 3 Laboratory of Biophysical Chemistry, Groningen Biomolecular Sciences and Biotechnology Institute, University of Groningen, Groningen, The Netherlands; 4 Mucosis BV, Groningen, The Netherlands; Institut Pasteur Paris, France

## Abstract

*Lactococcus lactis* expresses the homologous glucosaminidases AcmB, AcmC, AcmA and AcmD. The latter two have three C-terminal LysM repeats for peptidoglycan binding. AcmD has much shorter intervening sequences separating the LysM repeats and a lower iso-electric point (4.3) than AcmA (10.3). Under standard laboratory conditions AcmD was mainly secreted into the culture supernatant. An *L. lactis acmAacmD* double mutant formed longer chains than the *acmA* single mutant, indicating that AcmD contributes to cell separation. This phenotype could be complemented by plasmid-encoded expression of AcmD in the double mutant. No clear difference in cellular lysis and protein secretion was observed between both mutants. Nevertheless, overexpression of AcmD resulted in increased autolysis when AcmA was present (as in the wild type strain) or when AcmA was added to the culture medium of an AcmA-minus strain. Possibly, AcmD is mainly active within the cell wall, at places where proper conditions are present for its binding and catalytic activity. Various fusion proteins carrying either the three LysM repeats of AcmA or AcmD were used to study and compare their cell wall binding characteristics. Whereas binding of the LysM domain of AcmA took place at pHs ranging from 4 to 8, LysM domain of AcmD seems to bind strongest at pH 4.

## Introduction

Peptidoglycan, the major cell wall material of Gram-positive bacteria, is composed of chains of *N*-acetylmuramic acid and *N*-acetylglucosamine linked by means of β(1-4) glycosidic bonds. These bonds are cleaved by peptidoglycan hydrolases (PGH) during cell separation and cellular autolysis [8,16,25]. Impairment of cell separation activity of PGHs leads to long chains of cells in the lactic acid bacteria *Lactococcus lactis* and *Streptococcus thermophilus* [25,37,40]. Most PGHs are composed of at least two distinct domains, a cell wall-binding domain and a catalytic domain [25]. The cell wall-binding domains assist in adhering the enzymes to the murein layer while the catalytic domains cleave the cell wall. A tight interplay between both domains is essential for optimal PGH activity [42]. The (auto) lysis of the cells of lactic acid bacteria due to the action of its PGHs has been shown to be essential during cheese ripening for the release of intracellular proteins such as peptidases that contribute to flavor development [43].

The Lysin Motif (LysM) is one of the highly conserved cell wall-binding domains in many bacterial PGHs (PF01476) [2,8,25]. Individual LysM domains are formed by 1 to 6 LysM repeats that are connected by short non-homologous amino acid linkers consisting mostly of Ser, Thr, Asp and Pro residues [7,8,33,46-49]. LysM repeats consist of 44 to 65 amino acid residues and have been shown to specifically and non-covalently bind to peptidoglycan and to chitin, a polymer of *N*-acetylglucosamine [8,33,40,46,48]. Currently eight complete genome sequences of *L. lactis* are known [1,3,4,12,19,32,38,50]. Blast searches using the LysM domain sequences of the major autolysin AcmA [7] showed that each strain putatively expresses five proteins containing one or more LysM repeats at their C-terminus, except *L. lactis* CV56, UC509.9, CV56 and IL1403, which lack the gene for a homolog of the putative prophage endolysin (muramidase) Llmg_0851 while a TagH homolog is missing in the strains UC509.9 and SK11 (Table 1). Besides AcmA, a homologous protein named AcmD is putatively expressed in all strains [34]. In all cases the LysM repeats are separated by intervening sequences that are Ser/Thr rich, except in Llmg_0851. The intervening sequence between the two LysM repeats of this protein is as short as those in the LysM domain of AcmD, but they share no homology. The iso-electric points (pIs) of the LysM domains of these proteins vary from 3.8 to 10.3, which may reflect the conditions for strength of their binding to peptidoglycan (Table 1) [8].

**Table 1:** LysM domain-containing proteins of *L. lactis* MG1363^a^.

*Protein*	*Activity/role*	*Nr. LysM* ^*b*^	*Location* ^*c*^	*pI*	*pI LysM*
AcmA (Llmg_0280)	Glucosaminidase	3	Sec	10.3	10.0
AcmD (Llmg_0509)	Glucosaminidase^d^	3	Sec	4.3	4.2
Llmg_0851	Muramidase^d^	2	Sec	6.0	6.0
TagH (Llmg_1623)	Teichoic acid export ATP-binding protein	1	Mem	5.5	3.8
Llmg_0731	unknown	1	Mem	6.7	7.9

^a^ Homologs of all 5 proteins are present in the 8 fully sequenced *L. lactis* strains, except Llmg_0851, which is absent in strain UC509.9, CV56 and IL1403, and a TagH homolog is missing in UC509.9 and SK11

^b^ All LysM repeats are located at the C-terminus of the indicated proteins

^c^ Sec; Secreted according SignalP predictions, Mem; Membrane inserted according TMHMM predictions

^d^ Endolysin

*L. lactis*, the paradigm of the lactic acid bacteria, possesses 22 putative PGHs [28] of which four encode *N*-acetylglucosaminidases namely, AcmA, AcmB, AcmC, and AcmD [25,30,34,43]. They have a highly conserved Glu residue and a tetrad of Tyr, Ala, Thr and Asp amino acid residues forming the active site in the catalytic domain [16]. Mutational analysis of the active site of AcmA showed that Glu94 and Tyr191 are the crucial amino acid residues for catalytic activity of the enzyme [18]. AcmA has been shown to be involved in cell separation and cellular autolysis while AcmB only contributes to cellular autolysis [6,7,17,39,41]. HPLC and mass spectrometry analyses of muropeptides released from *B. subtilis* peptidoglycan with purified AcmA, AcmB or AcmC showed that all three lactococcal enzymes have *N*-acetylglucosaminidase specificity [16,17,41]. AcmD was hypothesized to also have *N*-acetylglucosaminidase activity, but this has not been confirmed experimentally [16]. Here the function of the AcmA homolog AcmD was investigated by analyzing an *acmD* mutant for cell separation, the effect of AcmD overexpression on autolysis and by examining AcmD substrate binding at different pHs. 

## Materials and Methods

### Bacterial strains, plasmids, growth conditions, and chemicals

The strains and plasmids used in this study are listed in Table 2. *L. lactis* was grown in M17 broth (Difco, Becton Dickinson, Le Pont de Claix, France) at 30° C as standing cultures or on M17 (1.5% w/v) agar, all of which were supplemented with 0.5% glucose (GM17). For the preparation of electrocompetent cells the media and agar contained 0.5 M sucrose (Acros Organics, Morris Plains, NJ). Erythromycin (Roche Diagnostics GmbH, Mannheim, Germany), chloramphenicol (Sigma Chemicals Co., St. Louis, Mo) and 5-bromo-4-chloro-3-indolyl-β-D-galactopyranoside (X-gal) (Sigma Chemicals Co.) were added to concentrations of 5 µg/ml, 5 µg/ml and 0.008%, respectively. *Escherichia coli* was grown in Tryptone Yeast (TY) extract medium (Difco, Becton Dickinson) at 37° C with vigorous agitation or on TY extract medium solidified with 1.5% (wt/vol) agar and containing 100 µg of erythromycin (Roche Diagnostics GmbH) per ml, when required. For *E. coli* EC101, 40 µg/ml kanamycin (Roche Diagnostics GmbH) was used and for *E. coli* MC1061 ampicillin (100 µg/ml) (Sigma, Zwijndrecht, The Netherlands) was used. All chemicals used were of analytical grade and, unless indicated otherwise, obtained from Merck KGaA (Darmstadt, Germany).

**Table 2:** Bacterial strains and plasmids used in this study.

*Strain or plasmid*	*Characteristic(s)* ^*a*^	*Source or reference*
MG1363	*L. lactis* subsp. *cremoris* ; Lac^-^ PrtP^-^; plasmid-free derivative of NCDO712	[13]
MG1363*acmAΔ1*	Derivative of MG1363 carrying a 701-bp *Sac*I-*Spe*I deletion in *acmA*	[7]
NZ9000	MG1363*pepN*::*nisRK*	[23]
NZ9000*acmAΔ1*	Derivative of NZ9000 with a 701-bp *Sac*I-*Spe*I deletion in *acmA*	[43]
NZ9700	Nisin-producer	[23]
LL108	MG1363 carrying multiple copies of the pWV01 *repA* gene in the chromosome	[27]
IL1403	*L. lactis* subsp. *lactis* ; plasmid-free	[10]
IL1403*acmA*::IS*S1*	Insertion of transposon IS*SI* in *acmA*	[40]
IL1403*acmA*::IS*S1acmD*::*myc*	Derivative of IL1403*acmA*::IS*SI* with an in-frame integrated *c-myc* epitope in *acmD*	This work
EC101	Km^r^; *E. coli* JM101 with *repA* from pWV01 integrated in the chromosome	[24]
MC1061	*E. coli araD139 Δ(araA-leu)7697 ΔlacX74 galK16 galE15(GalS) λ- e14- mcrA0 relA1 rpsL150(strR) spoT1 mcrB1 hsdR2*	[14]
pVE6007	Cm^r^, Ts derivative of pWV01	[31]
pORI*acmD*::*myc*	Em^r^, LacZ, pORI280*acmD* with *c-myc* epitope inserted into the *Bam*HI site	This work
pNG304	Cm^r^, pNZ8048 derivative containing the pre-pro sequence of *prtP* fused to *msa2* under control of P_*nisA*_	[41]
pNG3041	Cm^r^, pNZ8048 derivative containing the pre-pro sequence of *prtP* fused to *msa2* and the C-terminal domain of acmA under control of P_*nisA*_	[41]
pNG3042	Cm^r^, pNZ8048 derivative containing the pre-pro encoding sequence of *prtP* fused to *msa2* and the C-terminal domain encoding sequence of *acmD* under control of P_*nisA*_	[5]
pBAD	Amp^r^, carrying arabinose-inducible promoter P_*araBAD*_	[15]
pBADcLIC	Amp^r^, pBAD derivative carrying arabinose promoter and His_10_ tag	[14]
pBADcLIC-GFP	Amp^r^, pBAD derivative carrying arabinose promoter and *gfp* fused to the His_10_ tag encoding sequence	[14]
pBADcLIC-GFP-LysM_AcmD_	Amp^r^, pBADcLIC-GFP with sequence encoding the 3 LysM repeats of AcmD in *Swa*I site	This work
pBADcLIC-GFP-LysM_AcmA_	Amp^r^, pBADcLIC-GFP with sequence encoding the 3 LysM repeats of AcmA in *Swa*I site	[49]

a PrtP^-^ = proteolytically inactive

Ts = thermosensitive

Amp^r^, Cmr, Emr = resistance to ampicillin, chloramphenicol and erythromycin, respectively

### General DNA techniques and transformation

Molecular cloning techniques were performed essentially as described by Sambrook et al. (23). Genomic DNA of *L. lactis* was isolated according to the method of Leenhouts et al. [29]. Minipreparations of plasmid DNA from *L. lactis* were obtained by the alkaline lysis method as described by Seegers et al. [37]. Plasmid DNA was isolated at a large scale using a Nucleobond kit PC 100 (Machery-Nagel, Düren, Germany) as specified by the supplier. Restriction enzymes, T4 DNA ligase, and deoxynucleotides were obtained from Roche Diagnostics GmbH and were used according to the supplier’s instructions. Polymerase chain reactions (PCR) were performed in a Master cycler gradient (Merck KGaA) using Taq DNA polymerase or Expand DNA polymerase according to the instructions of the manufacturer (Roche Diagnostics GmbH). PCR products were purified using the High pure PCR product purification kit and protocol (Roche Diagnostics GmbH). *E. coli* and *L. lactis* were transformed by electroporation using a Gene pulser (Bio-Rad Laboratories, Richmond, CA) as described by Zabarovsky and Winburg [51] and Leenhouts and Venema [26], respectively. *E. coli* MC1061 cells were transformed with the recombinant vector by the heat-shock method [45].

### Construction of AcmA and AcmD fusion proteins

**Figure 1:**
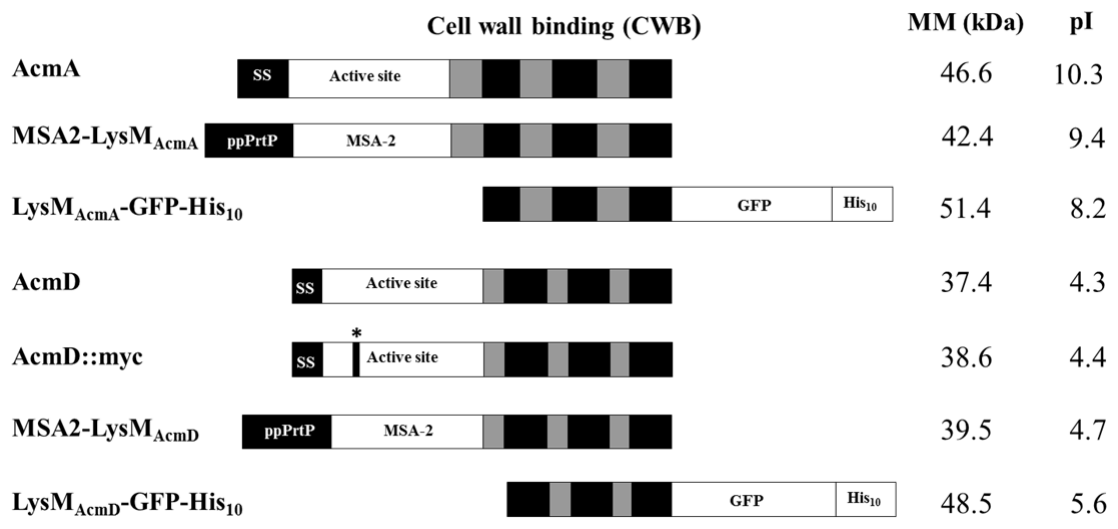
Modular architecture of AcmA, AcmD and the fusion proteins used in this study. The description of the protein is depicted on the left and their respective molecular masses (MM) (kDa) and pIs are given on the right. Dark and light grey boxes indicate the LysM repeats and their intervening amino acid sequences, respectively. *, site of in-frame myc-tag insertion in the active site of AcmD; MSA-2, *Plasmodium falciparum* Merozoite Surface Antigen-2; GFP, Green Fluorescence Protein; His_10,_ tag of 10 Histidine residues; SS, Signal Sequence; ppPrtP, pre-pro of prtP.

The modular architecture of all the constructs used in this study is depicted in Figure 1. Plasmid pNG*acmD* [41] was digested with the restriction enzyme *Bam*HI for which a recognition site is located in the active site of AcmD. The oligonucleotides pACMDmyc1 (GAT C T A G AACAAAAACTTATTTCAGAAGAAGATCTT, underlined *Xba*I) and pACMDmyc2 (GATCAA G A T C TTCTTCTGAAATAAGTTTTTGTTCT, underlined *Xba*I) were annealed at 70^o^C and cloned into the *Bam*HI site of pNG*acmD* resulting in the plasmid pNG*acmD::myc*.

Primers pACMD2 and pACMD3 (CGG A A T T CAAGGAGGAGAAATATCAGGAGG; underlined *Eco*RI site) were used to amplify a 603-bp fragment encoding the C-terminal domain of AcmD that contains the three LysM repeats, using chromosomal DNA of *L. lactis* IL1403 as a template. Upon digestion with *Eco*RI and *Hin*dIII the fragment was cloned into the *Eco*RI/*Hin*dIII sites of pNG3041 thereby replacing the 1162-bp domain encoding the LysM repeats of AcmA. The resulting plasmid was named pNG3042 [5].

The LysM-containing domains of AcmA and AcmD were amplified by PCR using the primers AcmA-LysMcLIC-forward (ATGGGTGGTGGATTTGCTG G A A A T A C T A A T T C T G G T G G C ) and AcmA-LysMcLIC-Reverse (TTGGAAGTATAAATTTTCT T T T A T T C G T A G A T A C T G A C C ) for AcmA and AcmD-LysMcLIC-Forward (ATGGGTGGTGGATTTGCTG T C G G A A C T T A T A A A G T A C A A G ) and AcmD-LysMcLIC-Reverse (TTGGAAGTATAAATTTTCA A T T T T A A T G G T T T G G C C T G G ) for AcmD. Nucleotide sequences of AcmA and AcmD LysM regions are in italic and underlined. LysM_AcmA_-GFP-His_10_ and LysM_AcmD_-GFP-His_10_ constructs were cloned in pBADcLIC-GFP plasmid containing Green Fluorescent Protein gene from *Aequorea* *victoriae* (Clontech, Mountain view, CA, USA), by the Ligation Independent Cloning procedure described by Geertsma et al. [14].

### Construction of an acmD insertion mutant of *L. lactis* IL1403

pNG*acmD::myc* was cut with *Sph*I and *Pst*I. The fragment carrying *acmD*::*myc* was inserted into the *Sph*I /*Pst*I sites of pORI280, an integration vector which lacks the gene encoding the replication initiation protein, *repA* [27,28]. The resulting plasmid, pORI*acmD*::*myc*, was obtained in *L. lactis* strain LL108, which carries multiple copies of the *repA* gene on the chromosome [27]. After transformation of *L. lactis* IL1403*acmA*::IS*S1* with pORI*acmD*::*myc*, erythromycin resistant integrants were checked by PCR with the primers: pEM280 (GCCCATATTTTTTCCTCC; annealing in the 5’-end of the erythromycin resistance gene) and pACMD2 (CGCA A G C T TCTGCAGAGCTCTTAGATTCTAATTGTTTGTCCTGG; underlined *Hin*dIII, which anneals to the 3’-end of *acmD*) as was described before [7].

Selection of the second crossover event was done as described by Leenhouts and Venema [26]. A 1040-bp region of *acmD* was amplified from the chromosomal DNA of selected integrants using the primers pACMD1 (CCTGTCATGAAACAGAAACATAAAT) and pACMD2, which anneal at either end of *acmD*. The presence of the c-myc epitope in *acmD* was confirmed by restriction with *Xba*I, as this site is only present in the DNA coding for this epitope. Although attempts were made to use the same approach to construct a single mutant of *acmD* in *L. lactis* IL1403, for unknown reasons this was not successful. Only first step integrants were obtained but excision resulted in all cases in reversion to the wild type.

### SDS-PAGE, zymography, Western hybridization and in-gel fluorescence detection

AcmA and AcmD activity was detected by a zymogram staining technique using SDS-polyacrylamide (PAA) (12.5%) gels containing 0.15% autoclaved, lyophilized *Micrococcus* *lysodeikticus* ATCC 4698 cells (Sigma Chemicals Co.), as described previously [7]. SDS-PAA gels without cells were stained with Coomassie brilliant blue (Bio-Rad Laboratories Inc). For Western hybridizations proteins were transferred from (2D)SDS-PAA gels to polyvinylidene difluoride membranes (Roche Diagnostics GmbH) as described by Towbin et al. [44]. MSA2-LysM_AcmA_ and MSA2-LysM_AcmD_ antigens were detected with a rabbit polyclonal anti-MSA2 antiserum [35] diluted 10000-fold and horseradish peroxidase (HRP)-conjugated goat anti-rabbit secondary antibodies (Pharmacia, Uppsala, Sweden). The c-Myc epitope was detected with 1:5000-diluted monoclonal mouse anti-c-myc antibodies (Amersham Biosciences, Piscataway, NJ) and 1:5000-diluted HRP conjugated anti-mouse antibodies. AcmA was detected with 1:5000-diluted polyclonal rabbit anti-AcmA active site antibodies [42] and 1:5000-diluted HRP conjugated anti-rabbit antibodies (GE Healthcare UK Ltd, Buckinghamshire, England). In all three cases, the Enhanced chemiluminescence detection system and protocol (Amersham Biosciences) was used.

LysM_AcmD_-GFP-His_10_ protein samples were subjected to SDS-PAGE with a 15% PAA gel and *in-gel* GFP fluorescence was visualized using a Gel Documentation System (Bio-Rad Laboratories Inc.). Bands obtained by Western hybridization were semi-quantified using the Quantity One program (Bio-Rad Laboratories B.V., Veenendaal, the Netherlands).

### Protein expression, isolation and purification

*E. coli* MC1061 bearing the desired plasmids (pBADcLIC-GFP-LysM_AcmA_ or pBADcLIC-GFP-LysM_AcmD_) were grown until an OD_600_ of 0.6-0.8 and induced with 0.2% arabinose (Merck KGaA) for 2 h. The cells were harvested at 5000 X g for 20 min at 4° C and the cell pellet was resuspended in lysis buffer (50 mM NaH_2_PO_4_ pH 8.0, 300 mM NaCl, 20 mM imidazole). Cell extract was obtained by sonication at 4° C using three cycles of 30 s pulses at 70% amplitude with 1 min intervals (Vibra Cell, Sonics & Materials, Newton, CT) followed by centrifugation at 20800 X g for 30 min at 4° C. LysM fusion protein was isolated and purified by Ni-NTA affinity chromatography as recommended by the manufacturer (Qiagen GmbH, Hilden, Germany).

### Two-dimensional (2D)-gel electrophoresis

Overnight cultures of *L. lactis* IL1403*acmA*::IS*S1* and IL1403*acmA*::IS*S1acmD*::*myc* were diluted to an OD_600_ of 0.05 in fresh GM17 medium and incubated at 30° C until an OD_600_ of 1.0 was reached. Subsequently, the cultures were centrifuged and proteins in the supernatant fractions were precipitated overnight at 4° C with TCA at a final concentration of 10%. Proteins were collected by centrifugation at 20000 X g for 20 min; the protein pellet was washed three times with acetone, air-dried and dissolved in a solution containing 8 M Urea, 2% CHAPS, 2 mM TBP, 0.2% ampholytes. The amount of protein loaded for 2D-gel electrophoresis was equal to that in 100 ml of supernatant fraction of GM17 cultures with an OD_600_ of 1.0. Isoelectric focusing (IEF) strips (Bio-Rad Ready Strips IPG; pH 4 to 7, 11 cm) were passively rehydrated overnight with the sample to be analyzed. IEF was performed with a Bio-Rad Protean IEF cell (Bio-Rad). The voltage was step-wise increased from 150 V (0.5 h) to 300 V (1 h) and 600 V (1 h), while the voltage was raised linearly to 8000 V until 25000 Vh. Subsequently, the strips were equilibrated twice for 15 min with 5 ml equilibration buffer (0.05 M Tris-HCl pH 8.8, 6 M Urea, 30% (w/v) glycerol, 2% SDS), the first time containing with 1% DTT, the second time with 4% iodoacetamide. The equilibrated strips were loaded on a standard SDS-(12%) PAA gel and run for 2.5 h at 100 V and 15^°^ C. The proteins in the gel were fixed by washing with a solution of 40% ethanol and 10% acetic acid. Staining was performed overnight with colloidal Coomassie brilliant blue (0.1% CBB G-250, 2% phosphoric acid, 20% ethanol); destaining was with distilled water. PDQuest 2D analysis software (Bio-Rad Laboratories Inc.) was used for comparison between gels and analysis of the 2D gel data.

### Bacterial growth, enzyme assays, and microscopy

OD_600_’s of cultures were measured in a Novaspec II spectrophotometer (Pharmacia Biotech AB, Uppsala, Sweden). To measure cellular lysis of *L. lactis*, cells were grown in GM17 at 30° C for 50 h. Subsequently, as a measure of the extent of culture lysis, release of intracellular X-prolyl dipeptidyl aminopeptidase (PepX) was measured using the chromogenic substrate Ala–Pro-p-nitroanilid (Bachem Feinchemicalien AG, Bubendorf, Switzerland) as described earlier [42].

The sample/substrate mixture was pipetted into a microtiter plate well, and color development was monitored in a THERMOmax microtiter plate reader (Molecular Devices Corporation, Menlo Oaks, CA) at 405 nm for 145 min at 37° C. The slope of the substrate hydrolysis/color development was calculated for two independent experiments.

Light microscopy pictures of *L. lactis* were made with a Zeiss microscope (Carl Zeiss, Thornwood, CA) and an Axiovision digital camera (Axion Technologies, Houston, TX). A fluorescence microscope (Zeiss Axiophot) fitted with a digital camera and a green filter was used to view GFP fluorescence.

For electron microscopic analysis, *L. lactis* MG1363 cells were treated with TCA (as described below) and incubated with MSA2, MSA2-LysM_AcmA_ or MSA2-LysM_AcmD_ as described above for untreated cells. The antibodies against MSA2 were diluted 1:1000 in PBS-containing 0.15 M glycine. Immunogold labeling was performed using Auroprobe 15 nm goat anti-rabbit IgG gold marker (Amersham Biosciences) using preparations of glutaraldehyde-fixed cells on Formvar-carbon-coated nickel grids. The labeled samples were stained with 0.1% uranyl acetate (w/v in water) and examined in a Philips CM10 transmission electron microscope at 100 kV.

To measure the influence of AcmD on cellular lysis, cells of *L. lactis* NZ9000 (pNG*acmD*) or NZ9000*acmAΔ1* (pNG*acmD*), both with or without nisin-induced AcmD, were mixed with AcmA and/or AcmD. Cells from 10 ml of culture of *L. lactis* NZ9000 (pNG*acmD*) or NZ9000*acmAΔ1* (pNG*acmD*), both either or not induced with nisin at an OD_600_ of 0.6, were collected by centrifugation at 5000 X g 2 h after induction. The cell pellets were resuspended in 10 ml of the supernatants of the *L. lactis* MG1363 (which contains both AcmA and AcmD) or *L. lactis* MG1363*acmAΔ1* (which contains AcmD) cultures and incubated at 30° C for 2.5 h (OD_600_ ~1.8). Subsequently, supernatant samples were collected and used to measure the extent of culture lysis by measuring PepX activity.

### Binding of fusion proteins to lactococcal cells

MSA2-LysM_AcmA_ and MSA2-LysM_AcmD_ binding studies were performed by mixing equal amounts of *L. lactis* cells (the amount of cells present in 1 ml of culture with an OD_600_ of 1.0) or with *L. lactis* cells that had been treated with trichloroacetic acid by boiling 25 µl of cell culture in 1 ml of 10% trichloroacetic acid (TCA) with 1 ml of supernatant of a nisin-induced *L. lactis* NZ9000 *acmAΔ1* (pNG3041, producing MSA2-LysM_AcmA_) or NZ9000 *acmAΔ1* (pNG3042, expressing MSA2-LysM_AcmD_) cultures. The suspensions were incubated at room temperature for 5 min, centrifuged (1 min at 20000 X g) and washed once with M17 broth. The pellets were subsequently resuspended in SDS sample buffer, boiled for 5 min, and subjected to SDS-PAGE.

Purified LysM_AcmA_-GFP-His_10_ and LysM_AcmD_-GFP-His_10_ purified proteins (3 µM each) were added to *L. lactis* cells in 50 mM NaH_2_PO_4_ (pH 4.0, 6.0 and 8.0), 50 mM NaCl buffer at room temperature and incubated for 30 min. Cells were centrifuged at 20000 X g for 1 min and the pellet was washed three times with the same buffer containing 150 mM NaCl to remove un-specifically bound proteins. Finally the washed pellet was resuspended in the same buffer and observed under a fluorescence microscope. To rule out the possibility of GFP interaction with *lactis* cells, GFP, purified by Hydrophobic Interaction Chromatography was used as a control. 

## Results

### Comparison of AcmA and AcmD

A comparison between AcmA [GenBank AAK04370] and AcmD [GenBank AAK04639] of *L. lactis* IL1403 was performed to pinpoint the differences between both proteins. Both have a similar modular structure (Figure 1): a signal sequence is followed by an active site domain (Glucosaminidase family, pfam PF01832) and a C-terminal LysM domain with three LysM repeats (pfam PF01476). The homology between the two proteins is around 58%. AcmD differs from AcmA in the length of the signal sequence (26 versus 57 amino acid residues), the presence of shorter amino acid sequences separating the repeats, while the protein has a pI of 4.3 instead of 10.3 for AcmA (Figure 1 and Table 1). His-tagged purified AcmD showed PGH activity only at pH 4 while AcmA is active at pH 4 to 8 [16,43].

AcmD of *L. lactis* subsp. *lactis* strains KF147 [38] and IL1403 [4] are identical while those of strains IO-1 [19] and CV56 [12] are 99% identical. The AcmD proteins of the *L. lactis* subsp. *cremoris* A76 [3], UC509.9 [1], MG1363 [50] and SK11 [32] are around 95% identical to IL1403 AcmD, respectively. The differences are mainly located between the active site domain and the LysM domain and in the intervening sequences separating the LysM repeats. No proteins homologous to AcmD that share the same low pI are encoded by the genomes of the other bacteria sequenced to date.

### AcmD contributes to cell separation and autolysis

A c-myc epitope was inserted in frame in the active site of AcmD to be able to follow expression and localization of the AcmD fusion protein and to make an *acmD* mutant by chromosomal integration. The chromosomal *acmD* gene in IL1403*acmA*::IS*S1* was replaced by *acmD*::*myc* via replacement recombination, resulting in an *acmAacmD* double mutant. However for yet unknown reasons the integration of *acmD*::*myc* into the genome of IL1403 failed. The effects of mutation of *acmD* on protein secretion by and autolysis and cell separation of *L. lactis* were investigated. Activity of AcmD could not be detected when cell-free extracts of *L. lactis* NZ9000, *L. lactis* IL1403, or IL1403*acmA*::IS*S1* all carrying an intact copy of the *acmD* gene in their chromosomes, were subjected to zymography using cell wall fragments of *M* *. lysodeikticus* or *L. lactis*, even after renaturation of the proteins in the gel at pH 4 (results not shown).

The protein patterns of culture supernatant fractions from the acmA and *acmAacmD* mutants did not differ significantly, as judged by 2D-gel electrophoresis. Two proteins were present in higher amounts in either the supernatant of the *acmA strain* or the *acmAacmD* double mutant while 4 proteins were detected only in the supernatant of the acmA mutant. The main difference was the expected shift in size of the secreted AcmD protein in the double mutant as a consequence of the c-myc epitope (from 34.9 to 36.6 kDa; Figure S1).

Cellular autolysis was examined by following the release of the cytoplasmic peptidase Pep-X into the culture supernatant [9]. No difference in lysis was observed between the *acmAacmD* double mutant and the *acmA* single mutant of *L. lactis* IL1403 (Figure S2).

**Figure 2:**
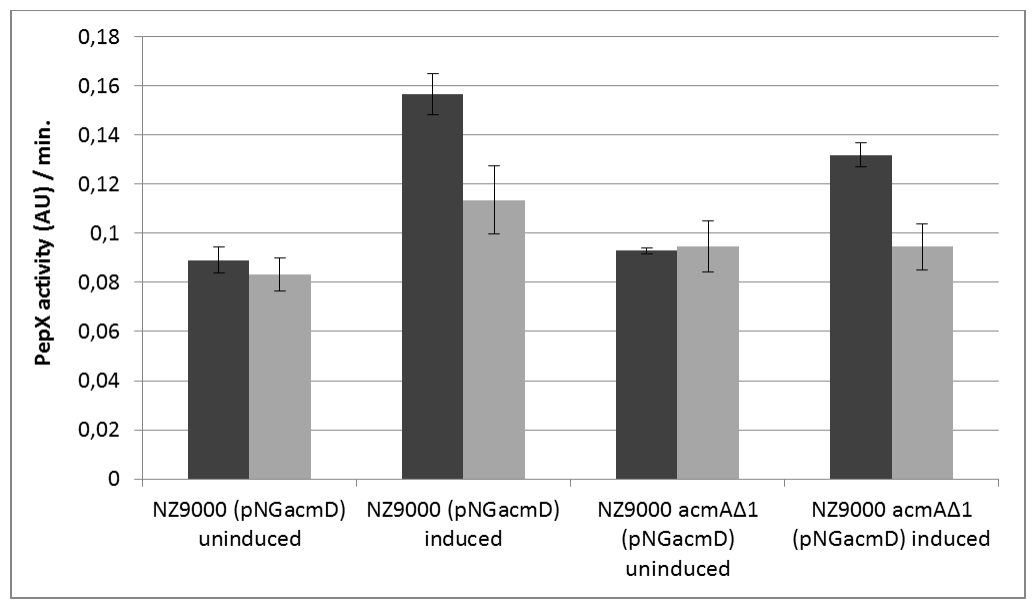
AcmD contributes to cell autolysis. Determination of cell lysis upon induced expression of AcmD. Cultures of *L. lactis* NZ9000 (pNG*acmD*) or *L. lactis* NZ9000*acmA∆1* (pNG*acmD*) were grown until an OD_600_ of 0.6 was reached. Both cultures were split in two. Expression of AcmD was induced in one half of each culture by the addition of nisin (10 ng/ml), the other halves were left un-induced. The cells of the four cultures were collected after 2 h and mixed with the cell-free culture supernatant of *L. lactis* MG1363 (dark grey bars) or *L. lactis* MG1363*acmA∆1* (grey bars). After 1 h incubation at 30° C, supernatants were analyzed for PepX activity (in arbitrary units (AU)). The slope of the substrate hydrolysis/color development was determined and is indicated as PepX activity (AU)/min. The average was calculated from two independent experiments.

Nisin-induced overexpression of AcmD has previously been shown to result in increased lysis of *L. lactis* MG1363 in the presence of AcmA while no additional lysis was obtained for the empty plasmid control [43]. Knowing that AcmD is secreted into the culture supernatant even though no activity could be detected, we investigated whether the contribution of AcmD activity to lysis occurs by peptidoglycan degradation during passage of the protein through the cell wall. Because a single *acmD* mutant could not be obtained the following approach was used to examine AcmD-mediated lysis: *L. lactis* strains NZ9000 (pNG*acmD*) and NZ9000*acmAΔ1* (pNG*acmD*) were grown in the presence of nisin to induce the expression of AcmD. Cell pellets were collected two hours after induction and resuspended in cell free supernatants of *L. lactis* MG1363, containing both AcmA and AcmD, or *L. lactis* MG1363*acmAΔ1*, containing only AcmD. Un-induced cells were used as controls. Using this approach the influence of AcmD when produced from the inside or added from the outside could be separately measured. The cells of the strains NZ9000 (pNG*acmD*) and NZ9000*acmAΔ1* (pNG*acmD*) released significantly more PepX and thus lysed to a larger extent when AcmD expression was induced (Figure 2, compare un-induced with induced). This was only the case when AcmA activity was present, upon suspension of the cells in the AcmA-containing MG1363 supernatant (Figure 2, see dark grey bars), showing that AcmD contributes to autolysis by its action within the cell wall.

**Figure 3:**
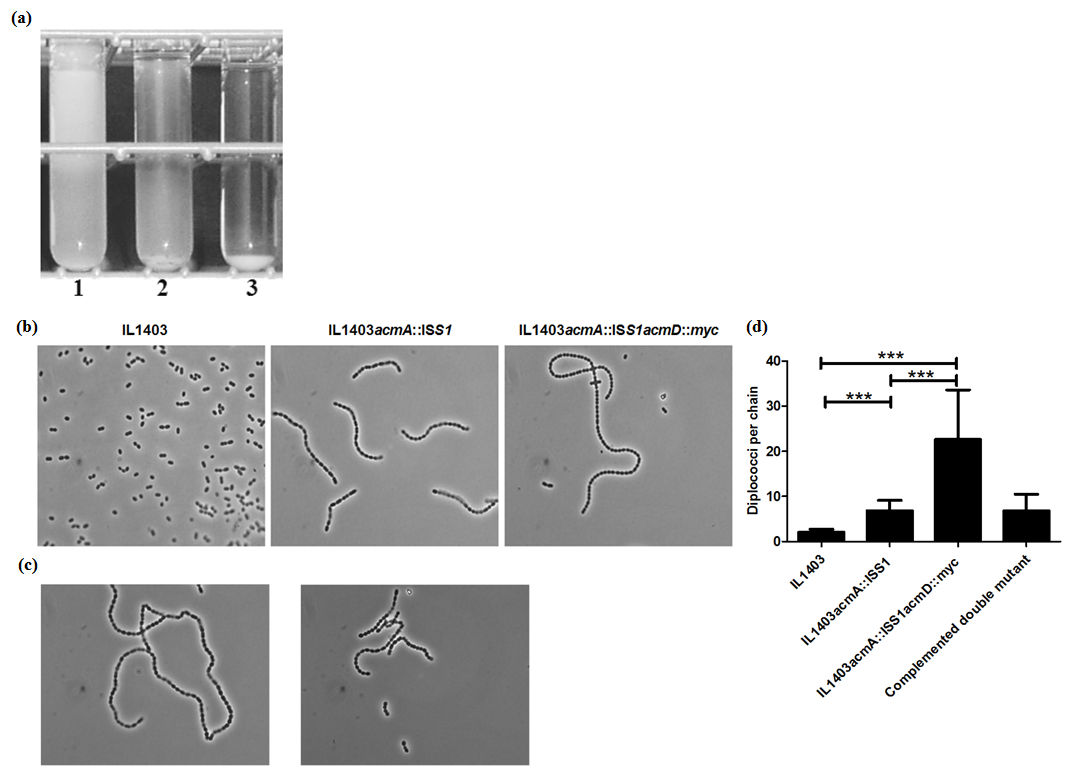
AcmD of *L. lactis* is involved in cell separation. (**a**) Turbidity and cell sedimentation of *L. lactis* IL1403 (1), IL1403*acmA*::IS*S1* (2) and IL1403*acmA*::IS*S1acm*D::*myc* (3) after overnight growth at 30° C in GM17 broth. (**b**) Light microscopic views of *L. lactis* IL1403, IL1403*acmA*::IS*S1* and IL1403*acmA*::IS*S1acmD*::*myc* after overnight growth at 30° C as standing cultures in GM17 medium. Magnification: 1250x in all three frames; representative views. (**c**) Phase contrast microscopic views of uninduced (left) and nisin (10 ng/ml)-induced (right) IL1403*acmA*::IS*S1acmD*::*myc* harboring plasmids pNGAcmD (AcmD) and pNZ9530 (nisRK). (**d**) Quantification of chain length in *L. lactis* IL1403, IL1403*acmA*::IS*S1*, IL1403*acmA*::IS*S1acm*D::*myc*, and nisin (10 ng/ml)-induced IL1403*acmA*::IS*S1acmD*::*myc* (pNGacmD) (the complemented double mutant). Number of diplococci per chain was counted and the mean of 30 chains were depicted. Standard deviation (***) indicates the significance as analyzed by Bonferroni’s multiple comparison test (*p*<0.05) using one-way ANOVA.

After overnight growth at 30° C in GM17 broth differences in cell sedimentation were observed between the *L. lactis* strains IL1403, IL1403*acmA*::IS*S1* and IL1403*acmA*::IS*S1acmD*::*myc*. The wild type IL1403 strain did not sediment whereas the acmA mutant did and the *acmAacmD* double mutant even more (Figure 3). Light microscopic analysis revealed that the latter strain formed very long chains in comparison to the other two (Figure 3B). The chains formed by the double mutant were calculated to be on average 2 to 3 times longer than those of IL1403*acmA*::IS*S1* (Figure 3D). Further, complementation of AcmD expression in the *acmAacmD* double mutant resulted in a decrease in the length of the chains to a length resembling that of the *acmA* single mutant (Figure 3C and D). This data indicates that AcmD, like AcmA, is involved in cell separation.

### LysM_AcmD_ binds to lactococcal cells preferably at low pH

**Figure 4:**
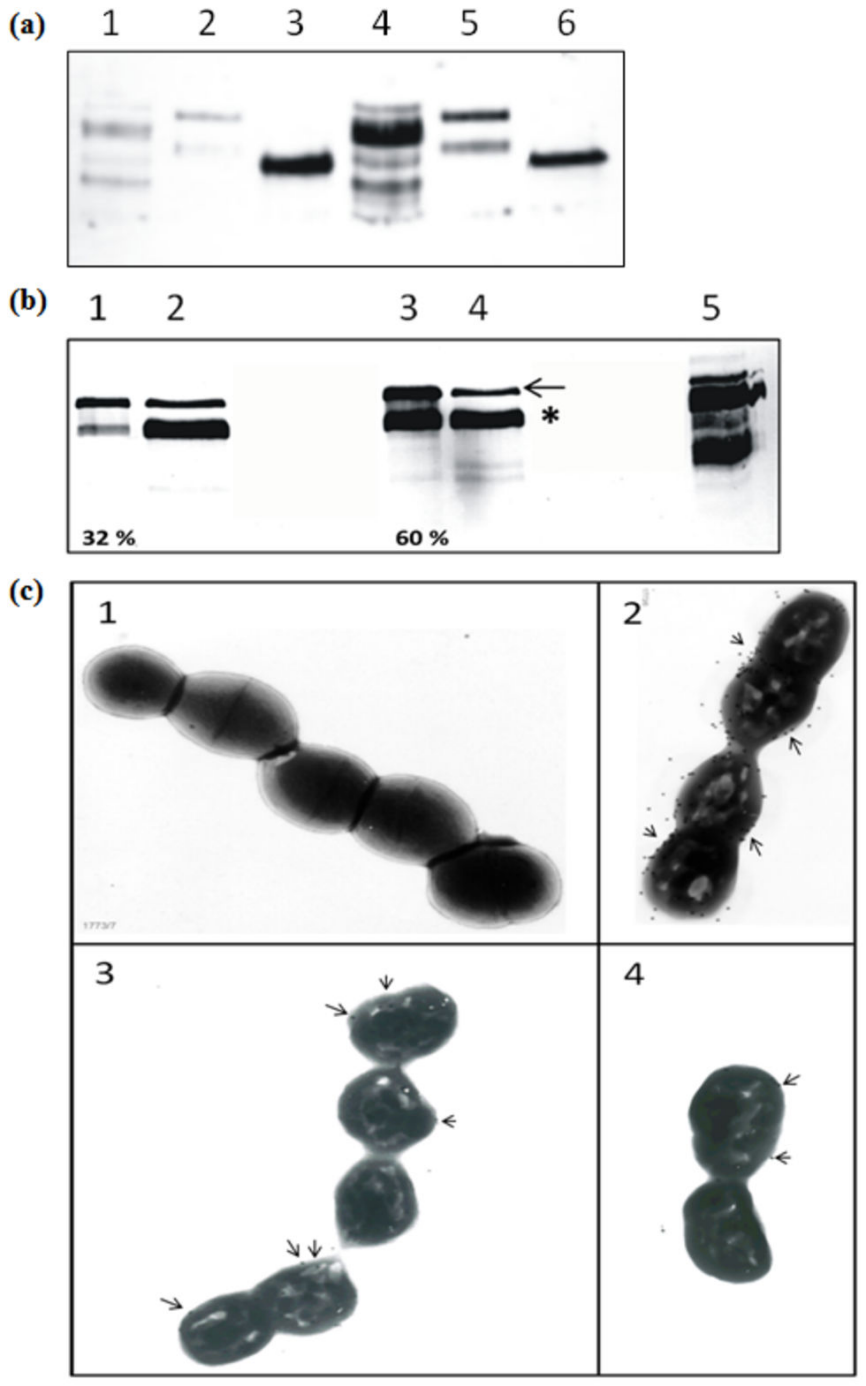
Binding of MSA2-LysM_AcmA_ and MSA2-LysM_AcmD_ to *L. lactis* cells. **a**. Anti-MSA2 antibody-treated Western blot showing binding of MSA2-LysM_AcmA_, MSA2-LysM_AcmD_ and MSA2 to non-treated (lanes 1, 2 and 3) and TCA-treated (lanes 4, 5 and 6) *L. lactis* cells, respectively. **b**. Western blot treated with anti-MSA2 antibody. Lanes 1 and 3 indicate MSA2-LysM_AcmD_ bound to TCA-treated *L. lactis* cells at pH 6.2 and 3.2, respectively. The unbound protein at pH 6.2 and 3.2 is shown in lanes 2 and 4, respectively. Lane 5, positive control: MSA2-LysM_AcmA_ bound to *L. lactis* at pH 6.2. Arrow points out Pro-MSA2-LysM_AcmD_ and * indicates mature MSA2-LysM_AcmD_. The percentage of both MSA2-LysM variants bound to *L. lactis* cells at pH 6.2 and 3.2 were semi-quantified (as the number of pixels present per mm^2^ of both bands in lanes 1 or 3) divided by the total signal of the cell and supernatant fractions (number of pixels per mm^2^ of all bands in lanes 1+2 or 3+4, respectively) is shown at the bottom of lanes 1 and 3. **c**. Transmission electron microscopic images of *L. lactis* incubated with MSA2 or MSA2 fusion proteins and subsequently incubated with rabbit anti-MSA2 antibodies and finally decorated with goat anti-rabbit IgG gold marker. Picture 1: non-treated *L. lactis* cells incubated with MSA2-LysM_AcmA_. Pictures 2, 3 and 4 show TCA-treated *L. lactis* cells incubated with MSA2-LysM_AcmA_, MSA2-LysM_AcmD_ and MSA2 proteins, respectively. Arrows indicate immunogold particles detected on the cells.

As peptidoglycan binding of AcmD is difficult to examine because its activity is not detectable and antibodies are not available, LysM_AcmD_ (pI=4.2, see column pI LysM in Table 1 and Figure 1) was fused to the MSA2 reporter protein, for which an antibody is available [35]. The fusion protein, MSA2-LysM_AcmD_, is secreted by fusing the signal sequence of the lactococcal proteinase PrtP to its N-terminal. Western detection revealed that MSA2-LysM_AcmD_ is secreted from *L. lactis* NZ9000*acmAΔ1* (pNG3042) upon nisin induction (results not shown). When a supernatant containing MSA2-LysM_AcmD_ was mixed with *L. lactis* MG1363 cells poor binding of the fusion protein was observed at pH 6.2 (Figure 4A). A similar result was obtained when *L. lactis* MG1363 cells were mixed with the supernatant of *L. lactis* NZ9000*acmAΔ1* (pNG3041), containing the MSA2-LysM_AcmA_ fusion protein [41]. Although previously we have shown that MSA2 alone cannot bind lactococcal cells [41] we did observe binding of the MSA2 protein in this study. When the binding of the three proteins was repeated with TCA-pretreated *L. lactis* cells a substantial increase in binding was observed for the MSA2-LysM_AcmA_ and MSA2-LysM_AcmD_ fusion proteins but not for the MSA2 protein (Figure 4A). To investigate whether the pI of MSA2-LysM_AcmD_ affects cell binding the binding to TCA-pretreated cells was repeated at pHs 3.2 and 6.2. Figure 4B clearly shows that MSA2-LysM_AcmD_ binds 2-fold more at pH 3.2 than at pH 6.2 (compare lanes 1 and 3). No detectable binding of MSA2-LysM_AcmA_ could be observed using electron microscopy (Figure 4C) while strong labeling signals was detected on TCA-treated cells. However, as for MSA2-LysM_AcmD_, only minor labeling signals were observed (Figure 4C). These results suggest that LysM_AcmD_ has poor cell wall binding properties around neutral pH (pH 7.2) while its binding to the cell wall is increased at a pH closer to its pI, when the net charge of the protein is positive.

### LysM domains of AcmD and AcmA bind differently to lactococcal cells

**Figure 5:**
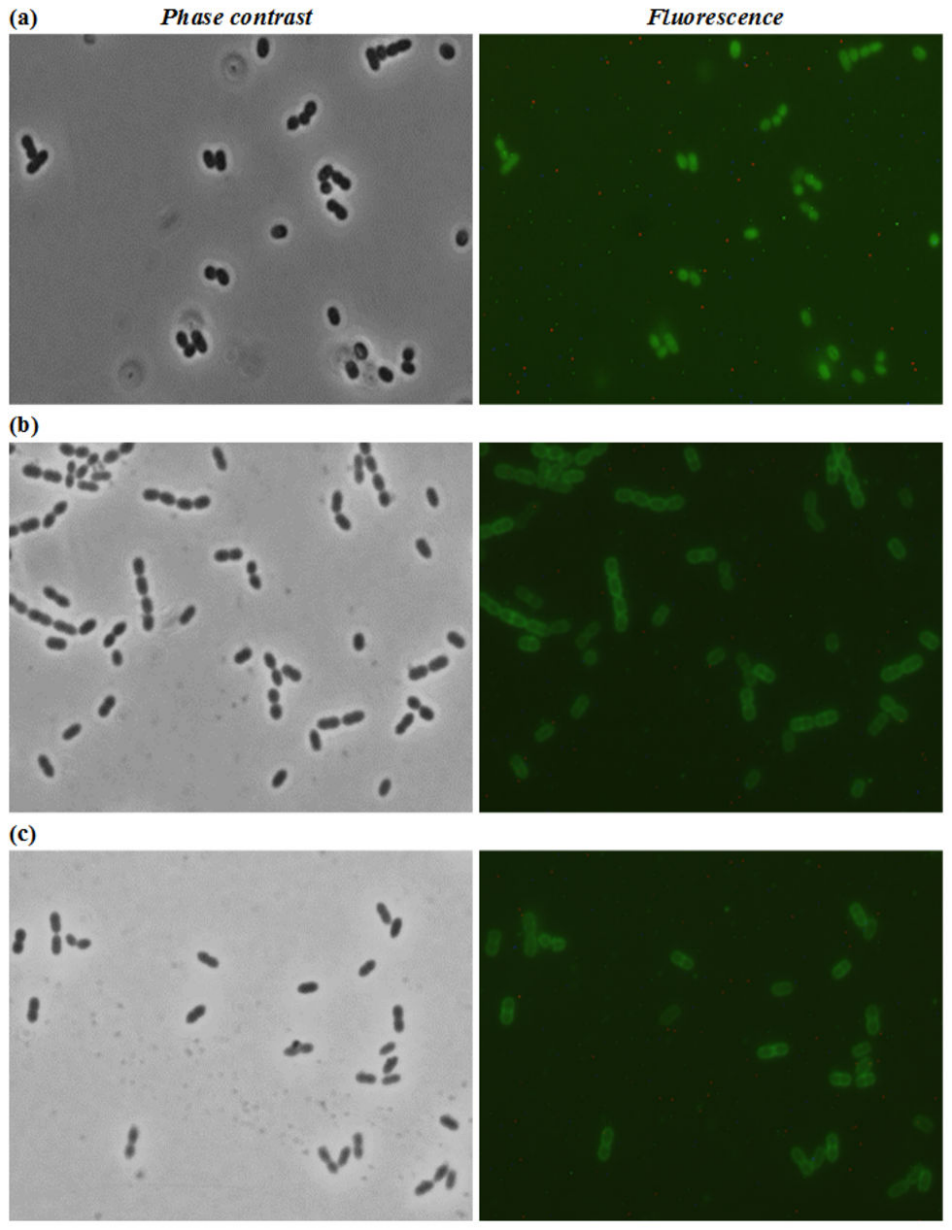
Binding of LysM_AcmD_-GFP-His_10_ and LysM_AcmA_-GFP-His_10_ to *L. lactis* NZ9000 cells. Phase contrast and fluorescence microscopy of *L. lactis* NZ9000 cells incubated with (**a**) LysM_AcmD_-GFP-His_10_ at pH 4.0 and (**b** and **c**) LysM_AcmA_-GFP-His_10_ at pH 4.0 and 8.0, respectively. Original magnification: 1250-fold in all frames.

The purified MSA2-LysM_AcmA_ fusion protein binds to the septum and poles of *L. lactis* cells when added from the outside to these cells [41]. Attempts to repeat these experiments with the MSA2-LysM_AcmD_ protein failed due to side effects of the immunodetection at pH 4. And because the MSA2 protein showed non-specific binding to *L. lactis* cells another approach was taken. To locate the sites of binding of LysM_AcmD_ on the cell, C-terminal GFP fusions of His_10_-tagged LysM_AcmA_ (pI=10, see column pI LysM in Table 1) and LysM_AcmD_ were expressed in and purified from *E. coli* using Ni-NTA. SDS-PAGE with a 15% PAA gel followed by *in-gel* fluorescence detection showed that the fusion proteins are of the proper size and fluoresce (results not shown and Figure S3). Purified LysM_AcmD_-GFP-His_10_ (48.5 kDa; pI=5.6) only binds to *L. lactis* NZ9000 cells at pH 4, not at pH 6 or pH 8 (Figure 5A and Figure S5). Phase contrast and fluorescence analyses showed that *L. lactis* does not intrinsically fluorescence under the conditions used (Figure S4A). HIC-purified GFP protein did not bind to the *L. lactis* cells under any of the conditions tested (Figure S4B and results not shown). Unlike the LysM_AcmD_ fusion protein, LysM_AcmA_-GFP-His_10_ (51.4 kDa; pI=8.2) bound to *L. lactis* cells at pH 4, pH 6 and pH 8 (Figure 5B and C and results not shown). 

## Discussion

In this study we show that the PGH AcmD of *L. lactis* is involved in cell separation as deletion of *acmD* results in increased length of the chains of cells. AcmD also contributes to autolysis, but only in the presence of AcmA. The LysM domain of AcmD binds more to the lactococcal cell wall at a pH close to its pI.

Under standard laboratory growth conditions at pH 6 to 7, AcmD is produced and mainly secreted by *L. lactis* (Figure S1). Peptidoglycan degrading activity could not be detected for the native expressed or overexpressed AcmD enzyme using a zymographic analysis method (results not shown). Earlier, His-tagged purified AcmD protein was shown to be active under renaturation conditions also employed in the present study (pH 4) [16]. A possible explanation for the different results in the two studies could be the very high protein concentration that was used in the earlier study. Although we were not able to show *in vitro* AcmD activity, a clear phenotypic effect was seen *in vivo*. The *L. lactis acmAacmD* mutant formed significantly longer chains (Figure 3B and D) and sedimented more readily than the wild type strain (Figure 3A) showing that AcmD is involved in cell separation, like its homolog AcmA. Complementation of the *acmD* mutation by plasmid-specified AcmD resulted in a decrease of chain length of the *acmAacmD* mutant (Figure 3C).

A second phenotypic effect was obtained upon overexpression of AcmD, namely increased autolysis of *L. lactis* (Figure 2). This effect was only observed when AcmA activity was also present: autolysis was not increased when AcmD was overexpressed in an AcmA-minus background. Although activity and presence in the cell wall of AcmD could not be unequivocally detected, it is possible that AcmD hydrolyses the peptidoglycan at specific sites during its passage through the cell wall. It has been shown that a pH-gradient is present over the cell wall of the Gram-positive model bacterium *Bacillus subtilis* and that this affects the activity of enzymes such as PGH’s [20]. Possibly, such a pH gradient also exists in the lactococcal cell wall, which would generate a local low pH required for AcmD activity.

The LysM_AcmA_-GFP fusion protein seems to bind at all pH’s tested to the whole surface of lactococcal cells (Figure 5B and C). Previously, hotspots for binding of the MSA2-LysM_AcmA_ fusion protein were detected by immunofluorescence microscopy [41]. This discrepancy may be caused by differences in the N- (MSA2) and C-terminal (GFP) fusion proteins used in the two studies e.g., the sizes of the proteins used, or by differences in the limits of detection of immunofluorescence and GFP fluorescence. Whereas the GFP fusion proteins can be detected in and (slightly) outside the cell wall, detection of the MSA2 fusion protein depends on proper outer surface exposure of the epitopes that are to be bound by the antibodies, which are too big to enter the cell wall [11]. LysM_AcmD_-GFP fusion protein (48.5 kDa; pI=5.6) binds to the cell wall at pH 4, when its net charge is slightly positive. At a pH above or below their pI, proteins carry either a net negative or positive charge, respectively, and local pH can greatly influence the characteristics of proteins or their domains. Although AcmA and AcmD share amino acid sequence homology in their active sites and their cell wall-binding domains, a significant difference exists in the pI values of their LysM domains. The observed differences in binding of the LysM domains of AcmA and AcmD (Figure 5) might be due to differences in the LysM domain pI’s and/or the intervening amino acid sequences in these domain. It has been suggested that the low pI of some of the LysM-containing proteins functions in binding of these enzymes to the cell wall at low pH or for positioning of active site domains at the proper location in the peptidoglycan matrix close to the membrane [8]. A more detailed study is needed to determine the specific binding sites in the cell wall peptidoglycan layer for the various types of LysM domains and whether the domains may be used to position different PGHs to different sites in the cell wall. 

## Supporting Information

Figure S1 **Comparison of 2D-gel images of supernatant fractions of *L. lactis* IL1403*acmA*::IS*S1* and *L. lactis* IL1403*acmA*::IS*S1acmD*::myc.** xmlns:xlink="http://www.w3.org/1999/xlink" xmlns:mml="http://www.w3.org/1998/Math/MathML">The amount of protein loaded in both cases was the equivalent of supernatant fraction of 100 ml of a GM17 culture with an optical density at 600 nm of 1.0. The position of the spots of the AcmD and AcmD::myc proteins, identified by Mass-spectroscopic analysis, and their molecular weights and pIs are indicated. Proteins that were more abundant in the supernatant fraction of IL1403*acmA*::IS*S1* (blue) or IL1403*acmA*::IS*S1acmD*::myc (red), and those unique in the supernatant of IL1403*acmA*::IS*S1* (yellow) or IL1403*acmA*::IS*S1acmD*::myc (green) are indicated. Sizes of the pre-stained molecular mass marker (kDa) are indicated in the middle.
(TIF)

Figure S2 **Deletion of *acmD* does not affect cell lysis during growth.** Release of intracellular X-prolyl dipeptidyl aminopeptidase (PepX) from *L. lactis* IL1403 (♦), IL1403*acmA*::IS*S1* (▲) and IL1403*acmA*::IS*S1acm*D::*myc* (■). Samples were taken at the indicated time points from the bacterial cultures incubated in GM17 broth. Upon removal of the cells by centrifugation the PepX-activity (in arbitrary units) released into the medium due to autolysis was determined using a chromogenic substrate, as described in the Materials and Methods section.
(TIF)

Figure S3 **Expression of LysM_AcmD_-GFP-His_10_ in *E. coli*.** Coomassie brilliant blue-stained SDS- (15%) PAA-gel (left) and *in-gel* GFP- fluorescence (right) showing the expression of LysM_AcmD_-GFP-His_10_ on SDS- PAGE with a 15% PAA gel. *E. coli* MC1061 bearing the pBADcLIC-LysM_AcmD_ was grown at 37° C until OD_600_ of 0.8 and induced with 0.2% arabinose for 2 h (see Materials and Methods section). The cell extracts of control and test samples were loaded on PAA gel for the identification of specific protein band. For the latter figure, the PAA gel is exposed to UV-light prior to coomassie staining for imaging the fluorescent bands. Prestained protein marker lane 1, cell extracts of empty vector control strain, un-induced control and 0.2%-arabinose induced test samples in lanes 2, 3 and 4, respectively. Arrows indicate LysM_AcmD_-GFP-His_10_ protein/activity bands.
(TIF)

Figure S4 **Negative and autofluorescence controls** Phase-contrast and fluorescence microscopy of *L. lactis* NZ9000 cells incubated at pH 4.0 with HIC-purified GFP (**a**) and without addition of any recombinant protein (**b**). Original magnification: 1250-fold in all frames.
(TIF)

Figure S5 **Binding of LysM_AcmD_-GFP-His_10_ to *L. lactis* NZ9000 cells at pH 6.0 and 8.0.** Phase-contrast and fluorescence microscopy of *L. lactis* NZ9000 cells incubated with LysM_AcmD_-GFP-His_10_ at pH 6.0 (**a**) and 8.0 (**b**). Original magnification: 1250-fold in all frames.
(TIF)
