# Supplementary figures and images for: AcmD, a Homolog of the Major Autolysin AcmA of Lactococcus lactis, Binds to the Cell Wall and Contributes to Cell Separation and Autolysis
Source: PLoS One. 2013 Aug 8;8(8):e72167. doi: 10.1371/journal.pone.0072167 (PMC3738550; doi:10.1371/journal.pone.0072167)

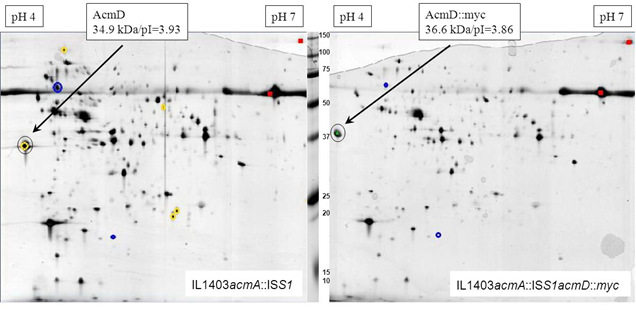

Supplement: Figure S1 — Comparison of 2D-gel images of supernatant fractions of L. lactis IL1403acmA::ISS1 and L. lactis IL1403acmA::ISS1acmD::myc. xmlns:xlink="http://www.w3.org/1999/xlink" xmlns:mml="http://www.w3.org/1998/Math/MathML">The amount of protein loaded in both cases was the equivalent of supernatant fraction of 100 ml of a GM17 culture with an optical density at 600 nm of 1.0. The position of the spots of the AcmD and AcmD::myc proteins, identified by Mass-spectroscopic analysis, and their molecular weights and pIs are indicated. Proteins that were more abundant in the supernatant fraction of IL1403acmA::ISS1 (blue) or IL1403acmA::ISS1acmD::myc (red), and those unique in the supernatant of IL1403acmA::ISS1 (yellow) or IL1403acmA::ISS1acmD::myc (green) are indicated. Sizes of the pre-stained molecular mass marker (kDa) are indicated in the middle. (TIF) [file pone.0072167.s001.tif]

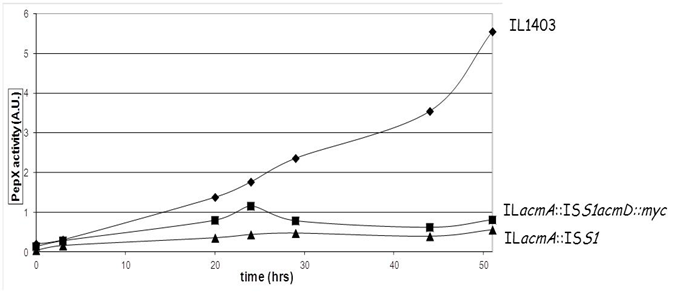

Supplement: Figure S2 — Deletion of acmD does not affect cell lysis during growth. Release of intracellular X-prolyl dipeptidyl aminopeptidase (PepX) from L. lactis IL1403 (♦), IL1403acmA::ISS1 (▲) and IL1403acmA::ISS1acmD::myc (■). Samples were taken at the indicated time points from the bacterial cultures incubated in GM17 broth. Upon removal of the cells by centrifugation the PepX-activity (in arbitrary units) released into the medium due to autolysis was determined using a chromogenic substrate, as described in the Materials and Methods section. (TIF) [file pone.0072167.s002.tif]

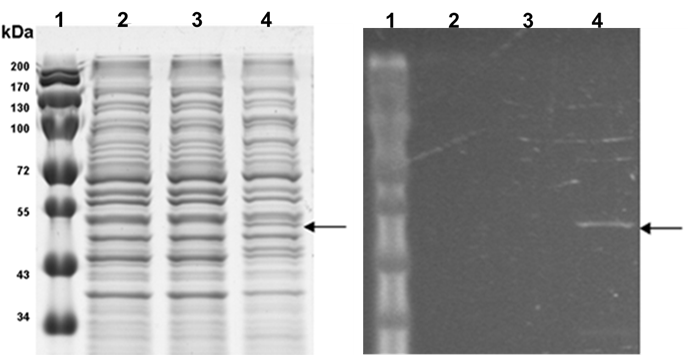

Supplement: Figure S3 — Expression of LysMAcmD-GFP-His10 in E. coli. Coomassie brilliant blue-stained SDS- (15%) PAA-gel (left) and in-gel GFP- fluorescence (right) showing the expression of LysMAcmD-GFP-His10 on SDS- PAGE with a 15% PAA gel. E. coli MC1061 bearing the pBADcLIC-LysMAcmD was grown at 37° C until OD600 of 0.8 and induced with 0.2% arabinose for 2 h (see Materials and Methods section). The cell extracts of control and test samples were loaded on PAA gel for the identification of specific protein band. For the latter figure, the PAA gel is exposed to UV-light prior to coomassie staining for imaging the fluorescent bands. Prestained protein marker lane 1, cell extracts of empty vector control strain, un-induced control and 0.2%-arabinose induced test samples in lanes 2, 3 and 4, respectively. Arrows indicate LysMAcmD-GFP-His10 protein/activity bands. (TIF) [file pone.0072167.s003.tif]

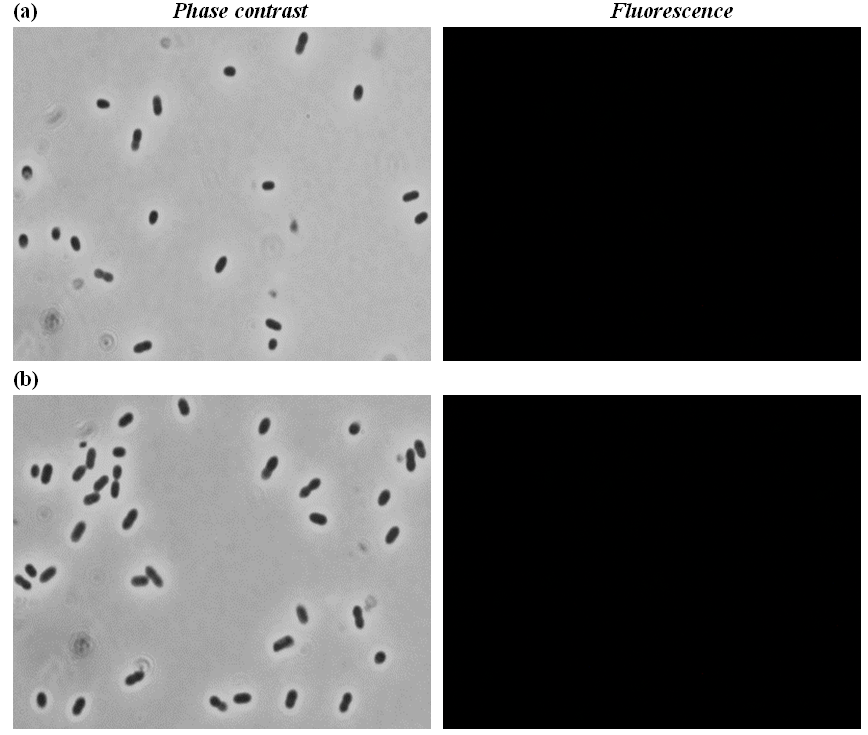

Supplement: Figure S4 — Negative and autofluorescence controls Phase-contrast and fluorescence microscopy of L. lactis NZ9000 cells incubated at pH 4.0 with HIC-purified GFP (a) and without addition of any recombinant protein (b). Original magnification: 1250-fold in all frames. (TIF) [file pone.0072167.s004.tif]

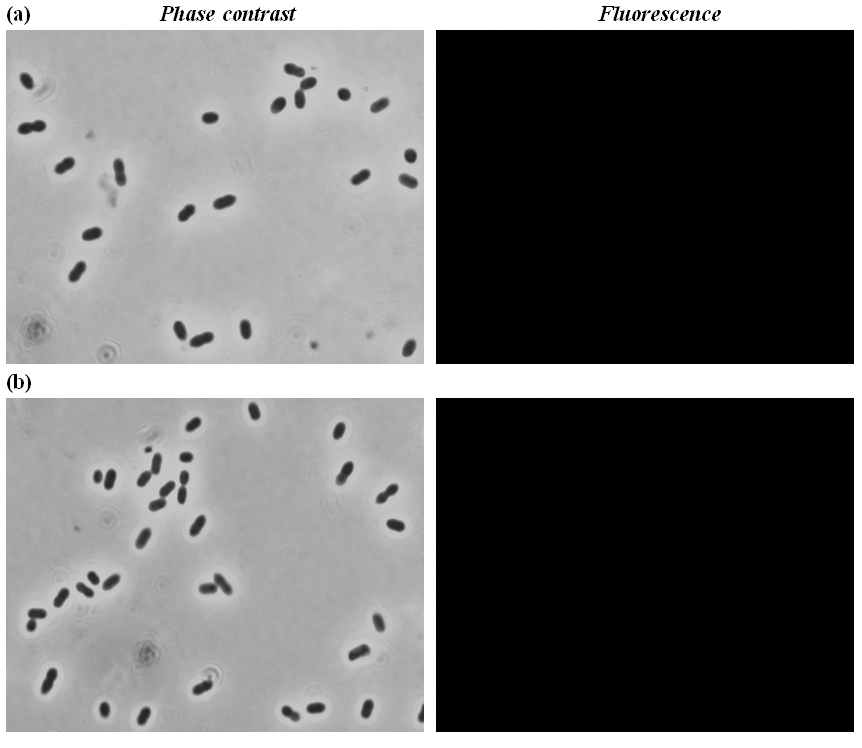

Supplement: Figure S5 — Binding of LysMAcmD-GFP-His10 to L. lactis NZ9000 cells at pH 6.0 and 8.0. Phase-contrast and fluorescence microscopy of L. lactis NZ9000 cells incubated with LysMAcmD-GFP-His10 at pH 6.0 (a) and 8.0 (b). Original magnification: 1250-fold in all frames. (TIF) [file pone.0072167.s005.tif]
